# Upregulation of autophagy by Ginsenoside Rg2 in MCF-7 cells

**DOI:** 10.1080/19768354.2018.1545696

**Published:** 2018-11-19

**Authors:** Yuheon Chung, Seula Jeong, Hyun Seok Choi, Seungil Ro, Jung Sup Lee, Jong Kun Park

**Affiliations:** a Division of Biological Sciences, Wonkwang University, Iksan, Republic of Korea; b Department of Physiology and Cell Biology, University of Nevada School of Medicine, Reno, NV, USA; c Department of Life Science, Chosun University, Gwangju, Republic of Korea

**Keywords:** Autophagy, Ginsenoside Rg2, UVB, MCF-7

## Abstract

Autophagy is a major intracellular degradation process that plays an important role in cell survival, stress responses, nutrient sensing and development. Our previous studies have shown that Rg2, a triterpenoid saponin contained in ginseng, protects cells against UVB-induced genotoxicity by increasing DNA repair, in possible association with modulation of protein levels involved in p53 pathway. In this study, we determined an upregulation of autophagy by Rg2. Rg2 treatment for 24 h in MCF-7, a breast cancer cell, did not show cytotoxicity up to 200 μM. Rg2 also upregulated the level of p-p53, p-AMPK, p-ACC, Atg-7 and LC3-II and decreased the level of p62 in concentration-dependent manners. We also determined the level of p53, AMPK, p62, Atg-7 and LC3 after UVB exposure and subsequent incubation in growth medium for 24 h. UVB increased the level of p-p53, p-AMPK, p-ACC and decreased the levels of p62, Atg-7 and LC3-II. Interestingly, Rg2 treatment for 24 h after UVB exposure increased the levels of p-p53, p-AMPK, p-ACC, Atg-7 and LC3-II and decreased the level of cyclobutane pyrimidine dimer, a UVB-induced DNA damage in concentration-dependent manners. All these results suggest that Rg2 increased autophagy and decreased UVB-induced DNA damage, in possible association with the modulation of protein levels in p53- and autophagic pathways.

## Introduction

Ultraviolet lights (UV) are divided into UVA, UVB, and UVC depending on wavelength, and each UV has different biological characteristics. The shorter the wavelength, the greater the amount of energy that destroys the molecular structure and has a critical effect on the cell. UVC (200–280 nm) is mostly absorbed in the ozone layer, and reaches to the surface of the earth in a very small extent. UVB (280–320 nm) and UVA (320–400 nm) reach the surface and show various biological effects (Liu et al. 2013). Exposure to UV causes various skin reactions such as erythema reaction, pigment reaction, skin aging and skin cancer. Recently, the amount of high energy UV reaching the surface is increasing due to ozone layer destruction (Matsumura and Ananthaswamy 2004, Kozma and Eide 2014).

UVB occupies 1–10% of the UV reaching the earth surface but is a more powerful injury source than UVA. Also, UVB has strong genotoxic and cytotoxic effects and acts mainly on the epidermal basal cell layer (Svobodová et al. 2012). UVB induces apoptosis with changes in the levels of apoptotic proteins (Bax, Bak, Bid) and anti-apoptotic proteins (Bcl-2 and Bcl-X) (Pustisek and Situm 2011). UVB acts on DNA and produces various damages such as cyclobutane pyrimidine dimer (CPD), 6-4 photoproduct (6-4PP) and 8-hydroxyguanine (8-OHdG) leading to gene mutations (Lo et al. 2005). Also, UVB induces oxidative stress through the enhancement of ROS and reduction of antioxidants (Aitken et al. 2007).

Rg2 is a triterpenoid saponin contained in ginseng. Other types of ginseng saponin include Rg1, Rb1, Rb2 and Rh1. Saponins have anti-apoptotic, anti-diabetic, antioxidant and neuroprotective effects. Rg2 binds to the glucocorticoid receptor (GR) as a glucocorticoid analogue and has a cellular mechanism similar to glucocorticoid. GR forms a dimer after binding to Rg2 and translocates to the nucleus (Nussey and Whitehead 2001). GR dimer binds to glucocorticoid receptor response elements (GREs) and transcriptionally activate other proteins such as p53 (Rhen and Cidlowshi 2005; Lu et al. 2006). Rg2 inhibits vascular cell adhesion molecule 1 and intercellular adhesion molecule 1, proteins produced by lipopolysaccharide (LPS), and blocks the degradation of IkB. It also inhibits Bax and caspase-3 and 9 which are increased by LPS, to exhibit anti-apoptotic functions (Fu et al. 2015). Rg2 activates antioxidative mechanism through the activation of Nrf-2 in neurons (Ye et al. 2016). Also, Rg2 reduces phosphoenolpyruvate carboxy kinase and glucose 6-phosphatase protein levels and inhibits glycosylation of hepatocytes in vitro through phosphorylation of AMP-activated protein kinase (AMPK) and glycogen synthase kinase 3 (Yuan et al. 2012). In HaCaT cells, the levels of superoxide dismutase and glutathione decreased after UVB irradiation, but Rg2 treatment increased in concentration-dependent manners (Kang et al. 2016). Rg2-induced autophagy through AMPK pathway *in vitro* and *in vivo* (Fan et al. 2017).

Autophagy is a mechanism of degradation of unnecessary or non-functional cellular components in cells (Klionsky 2008, Kobayashi 2015). The target components are surrounded by membranes to separate them from other components in the cell, which is called the autophagosome. Autophagosomes are combined with lysosomes to degrade and recycle intracellular substances (Mizushima and Komatsu 2011, Patel et al. 2012). Autophagy is largely divided into macroautophagy, microautophagy, chaperon-mediated autophagy (CMA) and mitophagy (Mizushima et al. 2002, Xie and Klionsky 2007, Mizushima et al. 2011, Youle and Narendra 2011, Lee et al. 2012). Macroautophagy can be induced for metabolic products, energy production for use in the biosynthetic process under stress conditions such as nutrients or energy deficiency (Levine et al. 2011). Microautophagy is a process in which unnecessary or non-functional cell organelles are degraded by the incorporation of lysosomes from the cytoplasm (Česen et al. 2012). In CMA, the non-functional component is transferred to the lysosome by the chaperone protein without formation of the vesicle (Levine et al. 2011). Mitophagy is the selective degradation of mitochondria by autophagy (Youle and Narendra 2011). Recent studies have shown that autophagy is associated with DNA repair. UVB-induced DNA repair was reduced when the twist-related protein 1 did not degrade in cells with inhibited autophagy (Qiang et al. 2016). Also, autophagy plays an important role in the progression of base excision repair and nucleotide excision repair (Zhang et al. 2015).

In the present study, we investigated whether the autophagy is upregulated through activation of AMPK, which is induced by Rg2-mediated p53 activation. We also determined the effect of autophagy on the UVB-induced DNA damage.

## Materials and methods

### Cell culture

MCF-7 (human breast cancer) cell line was purchased from Korea Cell Line Bank (Korea). Cells were cultured in RPMI1640 supplemented with 10% FBS (Wellgene, Korea), penicillin (100 U/mL) and streptomycin (100 µg/mL). The medium was adjusted to pH 7.2–7.4 with 10 mM HEPES (Sigma Aldrich, USA) and 0.37% sodium bicarbonate (Sigma Aldrich). 0.025% trypsin-EDTA (Wellgene) was used for subculture. Rg2 was treated after UV radiation.

### UV irradiation

A G15T8E UVB lamp (Sankyo, Japan) was used for UV irradiation. The dose of UV radiation was calibrated with UV radiometer (UVP, USA). Cells were treated with one or several concentrations of UV irradiation. The cells in a 90 mm plate were washed twice with phosphate-buffered saline (PBS) after the removal of media, and they were exposed to UVB radiation.

### Cell viability assay

Cell viability was measured by 3-[4,5-dimethylthiazol-2-yl]-2,5-diphenyl tetrazolium bromide (MTT) (Sigma Aldrich) assay. Cells were cultured in 96-well plates (SPL, Korea), exposed to UVB, and post-incubated with medium containing various concentrations of Rg2 for different time periods. The cells in each well were treated 20 µL of 5 mg/mL MTT solution and then incubated for 2 h at 37℃. Finally, each well was treated with DMSO and measured at 570 nm using a microplate reader (Retisoft Inc, Canada). The experiments were performed three times independently.

### Immunodot blot assay

For immunodot blot analysis, samples of 1 µg of DNA per dot were loaded on PVDF membranes (Milipore, Germany). After transfer, membranes were washed with TBS-T (TBS-0.05% Tween 20). After blocking with 5% skim milk, blots were incubated with anti-CPD (Cosmo Bio, Japan) in TBS-T for 12 h followed by washing for twice with TBS-T. Anti-mouse secondary antibody conjugated horseradish peroxidase was incubated for 1 h. After washing, the blots were treated with ECL plus (ELPIS, Korea), and chemiluminescence was detected by X-ray films. The experiments were performed three times independently.

### Western blot

Hot Laemmli lysis buffer supplemented with 1 mM PMSF (Sigma) was directly added to cells, and after centrifugation. Samples were boiled for 10 min, and then subjected to electrophoresis on sodium dodecyl sulfate-polyacrylamide gel. Samples were transferred to PVDF membranes and washed with TBS-T. After blocking with 5% skim milk, blots were incubated with anti-p53, anti-p-p53, anti-AMPK, anti-p-AMPK (Santacruz, USA), anti-p62 (Abcam, UK), anti-ACC, anti-p-ACC, anti-Atg-7, anti-AKT, anti-LC3 and anti-actin (Cell Signaling, USA) in TBS-T for 12 h followed by washing for twice with TBS-T. Secondary antibody conjugated horseradish peroxidase was incubated for 1 h. After washing, the blots were treated with ECL plus, and chemiluminescence was detected by X-ray films. The experiments were performed three times independently.

### Statistical analysis

Statistical analyses were performed using the Student’s *t*-test to determine whether the differences were statistically significant. The means of normalized values from at least three independent experiments were determined and subjected to Student’s *t*-test.

## Result

**Figure 1.**
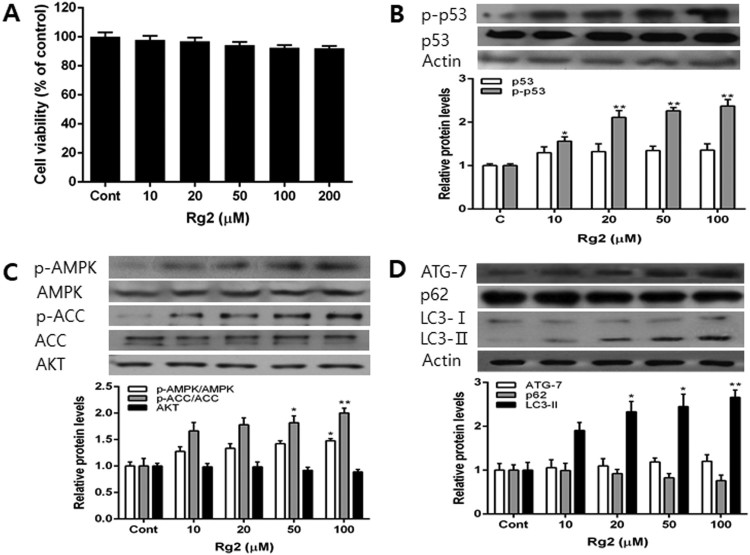
Effects of incubation for 24 h with various concentration of ginsenoside Rg2 on cell viability and the levels of p53, p-p53, AMPK, p-AMPK, ACC, p-ACC AKT, Atg-7, p62 and LC3 protein. Cells were incubated for 24 h in growth medium or medium containing various concentrations of Rg2. The cell viability was determined by MTT assay (A) and the levels of p53, p-p53, AMPK, p-AMPK, ACC, p-ACC, AKT, Atg-7, p62 and LC3 protein level were determined by western blot (B, C and D). Each data point represents the mean ± SD of triplicate experiments. *p < .05 and **p < .01, respectively, versus growth medium incubation (Cont).

Various concentrations of Rg2 were treated to confirm autophagy regulation by Rg2. MCF-7 cells were treated with Rg2 and the cell viability was determined by MTT assay. Rg2 did not show any cellular toxicity up to 200 µM (Figure 1(A)), and thus concentrations from 0 to 100 µM of Rg2 were employed in this study. MCF-7 cells were treated with Rg2 for 24 h, and p53 and p-p53 protein levels were measured by western blot. Rg2 significantly increased the p53 and p-p53 protein levels in a concentration-dependent manner as compared to the nontreated control group (Figure 1(B)). In order to determine whether Rg2 upregulated the levels of autophagy-related proteins, we measured the protein levels of AMPK, p-AMPK, p62, Atg-7 and LC3. The levels of AMPK, p-AMPK, p-ACC, Atg-7 and LC3-II increased and those of AKT and p62 decreased by Rg2 (Figure 1(C and D)). Rg2 treatment increased Atg-7 and LC3-II and decreased p62 (Figure 1(D)). These results suggest that Rg2 treatment increases the autophagy in MCF-7 cells.

**Figure 2.**
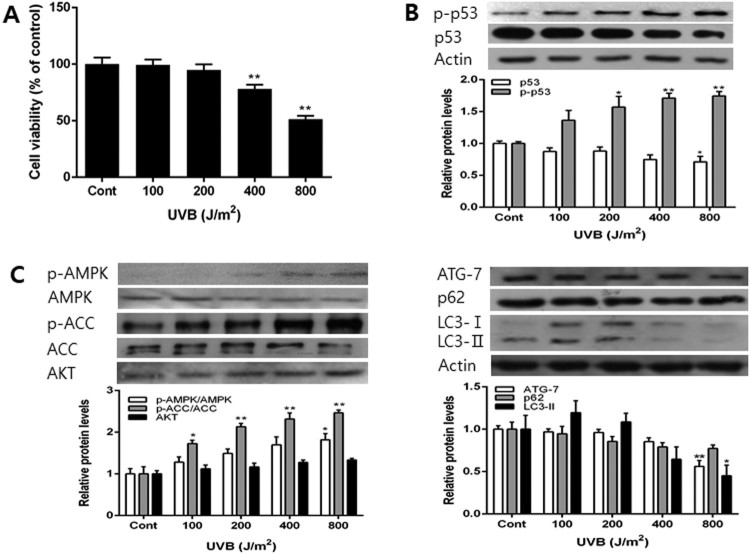
Effects of incubation for 24 h with various doses of UVB on the cell viability and the levels of p53, p-p53, AMPK, p-AMPK, ACC, p-ACC, AKT, Atg-7, p62 and LC3 protein. Cells were incubated for 24 h in growth medium after various doses of UVB exposure. The cell viability was determined after MTT assay (A) and the levels of p53, p-p53, AMPK, p-AMPK, ACC, p-ACC, AKT, Atg-7, p62 and LC3 protein were determined by western blot (B, C and D). Each data point represents the mean ± SD of triplicate experiments. *p < .05 and **p < .01, respectively, versus growth medium incubation (Cont).

Various doses of UVB were irradiated to determine the changes of in the UVB-induced autophagy. Cell viability decreased in a UVB dose-dependent manner. We determined the IC50 value to 800 J/m^2^ (Figure 2(A)). UVB exposure increased the level of p-p53 and decreased p53 level in dose-dependent manners (Figure 2(B)). The decrease of p53 is thought to be due to the increase in the phosphorylated form of p53. UVB exposure also induced the increases in p-AMPK, p-ACC and AKT levels (Figure 2(C)). As the dose of UVB increases, the levels of Atg-7, p62 and LC3 decreased, similarly to the earlier reports (Zhang et al. 2015). However, low doses of UVB-induced little changes in Atg-7, while increased and the level of LC3-II. Doses above 200 J/m^2^ reduced the levels of p62 and LC3-II (Figure 2(D)). The protein degradation seems to be induced by autophagy at doses above 200 J/m^2^.

**Figure 3.**
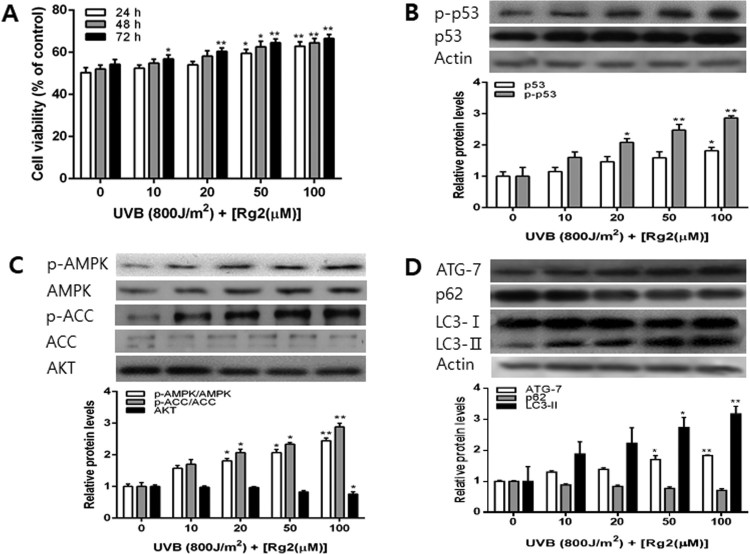
Effects of post-incubation for 24 h with various concentrations of Rg2 on the cell viability and levels of p53, p-p53, AMPK, p-AMPK, ACC, p-ACC, AKT, Atg-7, p62 and LC3 protein in UVB-exposed MCF-7 cells. Cells were post-incubated for 24 h in growth medium or medium containing various concentrations of Rg2 after UVB exposure. The cell viability was observed after MTT assay (A) and the levels of p53, p-p53, AMPK, p-AMPK, ACC, p-ACC, AKT, Atg-7, p62 and LC3 protein level were determined by western blot (B, C and D). Each data point represents the mean ± SD of triplicate experiments. *p < .05 and **p < .01, respectively, versus growth medium incubation.

After 800 J/m^2^ UVB exposure, various concentrations of Rg2 were post-incubated and the cell viability and autophagy regulation were determined. Cell viability increased in an Rg2 concentration and time-dependent manners. Cell viability was highest at 100 µM Rg2 which caused about 35% increase in the cell viability as compared to growth medium (Figure 3(A)). After UVB irradiation, the levels of p53 and p-p53 increased in Rg2-treated MCF-7 cells for 24 h (Figure 3(B)). The levels of AMPK, p-AMPK and p-ACC increased and the level of AKT decreased (Figure 3(C)). After UVB exposure, Rg2 increased levels of the autophagy-related proteins such as Atg-7 and LC3-II in concentration-dependent manners (Figure 3(D)). Rg2 treatment after UVB exposure showed that the level of p62 decreased (Figure 3(D)). These results suggest that the combined treatment of UVB and Rg2 synergistically upregulated the levels of p-p53 and LC3-II, an autophagy-related protein.

**Figure 4.**
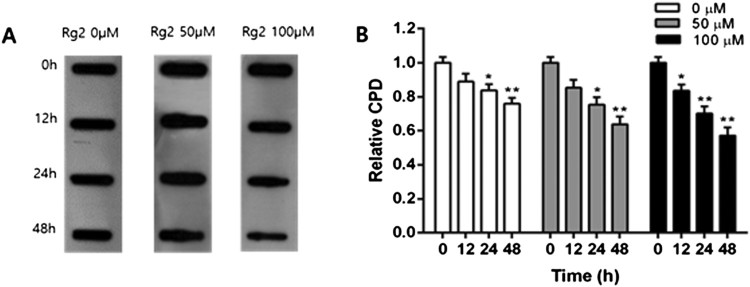
Effects of post-incubation for different time periods with various concentrations of Rg2 on the levels of CPD in UVB-exposed MCF7 cells. Cells exposed to 800 J/m2 UVB were post-incubated for various time periods in normal growth medium or medium containing Rg2. CPD level was determined by immunodot blot. Each data point represents the mean ± SD of triplicate experiments. *p < .05 and **p < .01, respectively, versus growth medium incubation.

The level of CPD, a UVB-induced DNA damage, was measured by immunodot blot in MCF-7 cells. In these experiments, 100 and 50 µM of Rg2 concentrations, in which p62 and LC3-II levels relatively increased, was selected. After UVB exposure, the level of CPD decreased in a time-dependent manner in growth medium and decreased about to 15% at 24 h as compared to 0 h. The level of CPD more decreased in a group treated with 50 µM (gray bar) than growth medium (0 µM, white bar) and decreased about to 20% as compared to the cells incubated in the growth medium. Also, the level of CPD more decreased by 100 µM (black bar) than 50 µM and decreased about to 24% at 48 h as compared to growth medium (Figure 4). The present results are consistent with the previous studies which showed that DNA repair is accelerated by p-p53 activated by Rg2 (Jeong et al. 2007, Ha et al. 2010).

**Figure 5.**
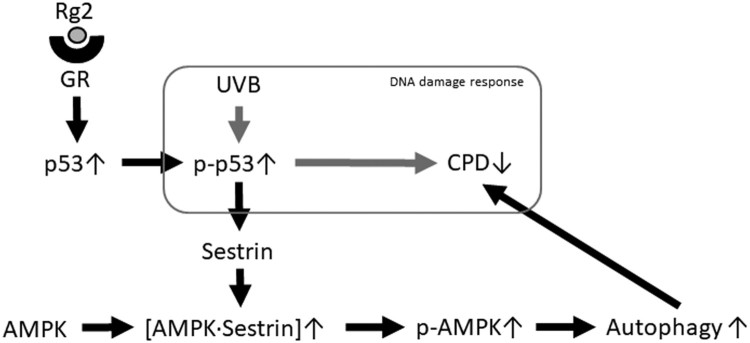
The role of proteins in autophagy upregulation. Rg2 induces autophagy involving p53, sestrin and AMPK. There is crosstalk among the pathways involving many of these critical signaling proteins.

The results obtained in the present study show that Rg2 treatment after UVB increases the level of p53, p-p53, p-AMPK and autophagy-related proteins and decreased the level of CPD induced by UVB. UVB-induced DNA damage is known to be decreased by p53 and autophagic mechanism (Verschooten et al. 2006, Zhang et al. 2015, Qiang et al. 2016). Our suggestion to these observations is presented in Figure 5: GR binds to Rg2 to form a dimer and translocate to the nucleus. In the nucleus, the dimer increases the level of p53 through transcriptional activation. When DNA damage agents like UV is treated, the level of p-p53 increases (Crightom et al. 2006). Activated p53 induces transcriptional activation of sestrin and increase in the nuclear level of sestrin. After AMPK interact with sestrin, it is activated by AMPK kinase. And the latter phosphorylates and activates sirtuin1 (Feng et al. 2007, Chang et al. 2015). These processes activate ATG proteins, cleave LC3 and increase the formation of autophagosome. The results of this study suggest that Rg2-induced autophagy accelerates the reduction of UVB-induced DNA damage such as CPD.

## Discussion

Saponin is a substance contained in various plants and some echinoderms. It is a generic name of a substance which is a glycoside of a steroid and triterpenoid saponin. Various saponins have different functions and toxicity. To use saponin, sufficient research should be conducted to verify the reliability and effectiveness.

Autophagy is known as part of the energy-producing process to survive. In addition, it was recognized as a process for large degradation of unnecessary proteins in cells. Recently, it has attracted attention as an important function in intracellular metabolism. The AMPK pathway and AKT/mTOR pathway are known to be the most directly regulating protein mechanisms of autophagy. Development of anticancer drugs agents that regulate autophagy by activating AMPK pathway or inhibiting AKT/mTOR pathway is underway (Chang et al. 2015). Autophagy can induce a powerful function when the anticancer drug is treated.

Autophagy is modulated by phosphorylated AMPK and p53. p62 and LC3-II regulated by phosphorylated AMPK and p53. Autophagy and p-p53 level were upregulated by *L.muscari* saponin in lung cancer cells. We suggest that Rg2 increase the level of p53 by transcriptional activation GR (Figure 1(B)). Increased autophagy by p53 is regulated by activating tuberous sclerosis complex1 (TSC1) and TSC2 and inhibiting mTOR pathway through activation of AMPK (Feng et al. 2007). Another mechanism of p53-autophagy is damage-regulated autophagy modulator (DRAM). DRAM interacts with p53 and binds to the lysosomal membrane (Crighton et al. 2006). According to the global genomic profiling, p53 transactivate autophagy-related proteins such as a member of serine/threonine-protein kinases Unc-51 like autophagy activating kinase 1 (ULK1), ULK2, a member of the DNA damage sensor UV radiation resistance-associated gene protein (UVRAG), Atg-2b, Atg-4a, Atg-4C, Atg-7, Atg-10, transmembrane protein 49 (Tmem49/Vmp1) (Kenzelmann Broz et al. 2013). Ei24, a gene that induces apoptosis by DNA damage, is known to be a p53-induced gene 8 (PIG8) and has recently been demonstrated as a new essential factor in autophagy induced by p53 (Zhao et al. 2012).

GR-activated p53 activates sestina/AMPK complex through transcriptional activation and induce AMPK activation by AMPK phosphorylation, thereby enhancing autophagy (Herold et al. 2002). In the present study, treatment with Rg2 alone or after UVB increase the levels of p-p53, p-AMPK, and LC3-II, a marker for autophagosome formation, which decreased p62, a marker for autophagic protein degradation (Figures 1C, D, 3C and D). Also, we determined CPD reduction in Rg2-treated MCF-7. Our previous study has shown that Rg2 increase p-p53 and decrease CPD in UVB-exposed HaCaT cells (Ha et al. 2010). Also, The DNA damage-induced transcription of this gene is mediated by both p53-dependent and p53-independent mechanisms (Ha et al. 2016).

The Rg2-activated GR transcriptionally activates p53, and AMPK is phosphorylated by p53, and activates the AMPK pathway (Figures 1B and 3B) (Feng et al. 2007, Chang et al. 2015). p53 is considered to be a major protein in the mechanism of GR signaling to AMPK (Figures 1C and 3C) (Kenzelmann Broz et al. 2013). The AMPK pathway and autophagy are also reported to be inhibited by PI3K/AKT/mTOR pathway. In addition, the autophagy is shown to improve DNA repair indirectly by degrading the TWIST1 protein which inhibits DNA repair (Qiang et al. 2016). The mechanisms by which autophagy is activated by Rg2 and associated with the enhanced DNA repair is not well known and further studies including siRNA and immunofluorescence in time course experiments should be conducted.
